# Franck-Condon Factors to High Vibrational Quantum Numbers III: CN

**DOI:** 10.6028/jres.068A.005

**Published:** 1964-02-01

**Authors:** R. W. Nicholls

## Abstract

Franck-Condon factor arrays have been computed numerically to high vibrational quantum numbers for the red (A^2^II_i_–X^2^Σ^+^) and violet (B^2^Σ^+^ – X^2^Σ^+^) band systems of CN.

## 1. Introduction

The CN molecule is a significant contributor to the spectra of comets and stellar atmospheres. It also plays an important rôle in many aspects of combustion spectroscopy. The red and violet band systems are its two most important electronic transitions. Definitive vibrational assignments and molecular constants exist for them [Douglas and Routly 1954]. Intensity measurements have been made on both systems and interpreted in terms of electronic transition moment variation across them and of smoothed band strengths [Ornstein and Brinkmann 1931; Nicholls 1956; Dixon and Nicholls 1958; Ferguson 1963]. Franck-Condon factor arrays are necessary parameters for these studies. The purpose of this paper is to supplement the limited and approximate arrays of Franck-Condon factors previously available for the systems [Fraser, Jarmain, and Nicholls 1954; Nicholls, Fraser, and Jarmain 1959].

In previous papers of this series, the straightforward numerical method of computation for Morse molecules to high vibrational quantum numbers have been described. [Nicholls 1960, 1961] The method has since been used on a number of important band systems and excitation transitions (Nicholls 1962a, b, c, d).

The computations were performed upon the IBM 7090 computer of the National Bureau of Standards using a program written by Miss R. Zucker of the Computation Laboratory.

## 2. Basic Data

The input data for the program are ω*_e_*, ω*_e_x_e_, r_e_*, *μ_A_* and υ_max_ for both states of the transition involved. These data were obtained from the work of Douglas and Routly and are listed in table 1.

## 3. Results

**Figure 1:**
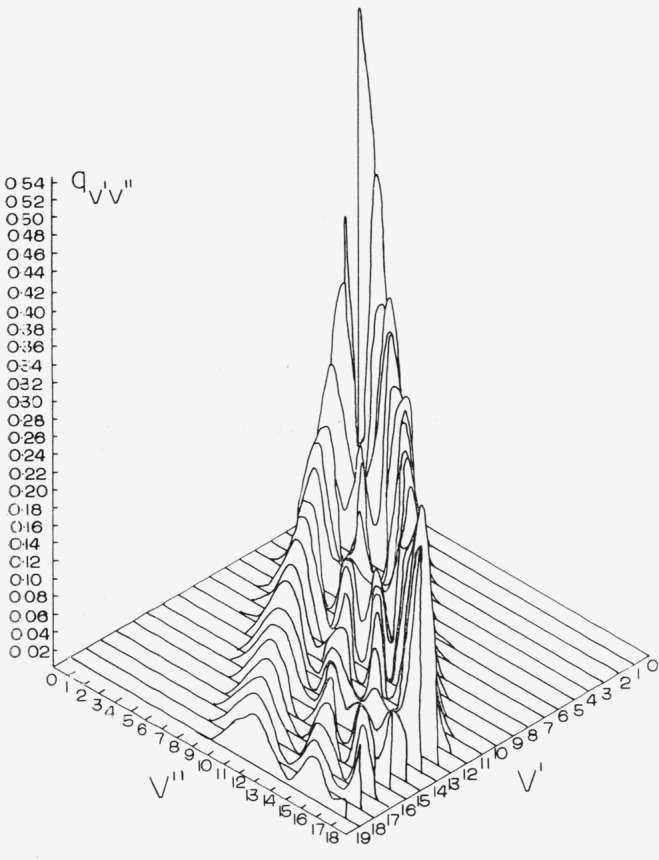
Franck-Condon factor surface for the *CN* red system.

**Figure 2:**
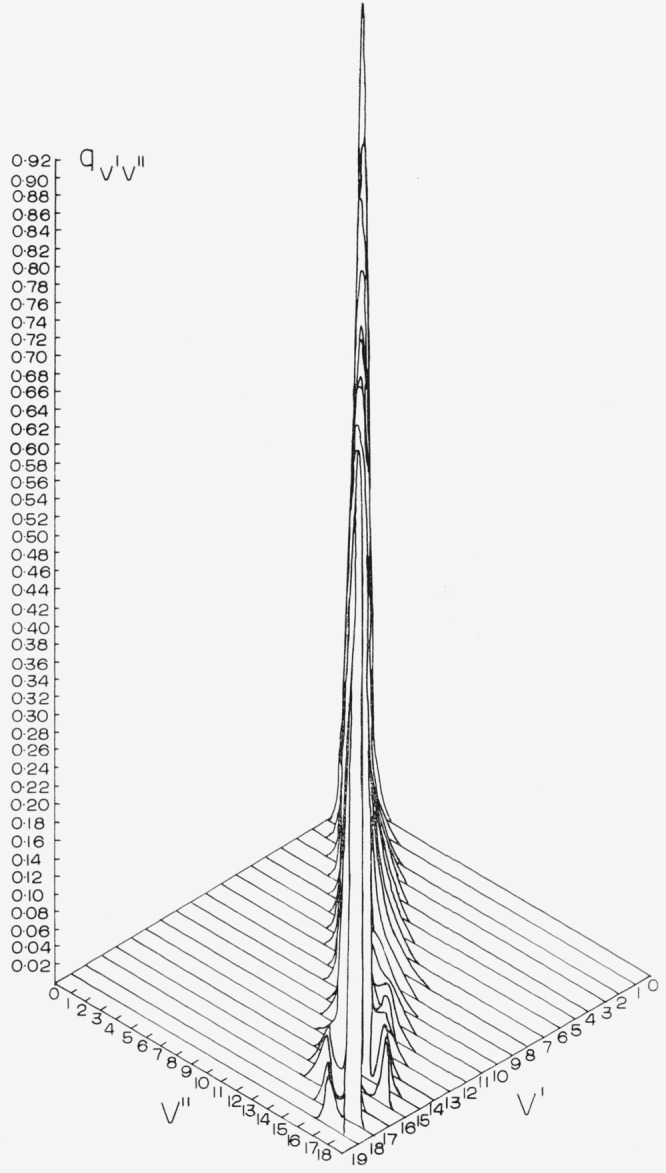
Franck-Condon factor surface for the *CN* violet system.

The Franck-Condon factor arrays for the CN red and violet systems are displayed in tables 2 and 3. Franck-Condon factor surfaces of these arrays are shown in figures 1 and 2. The strongly developed Condon loci are clearly evident in both cases. The CN violet system, having a very small Δ*r_e_* (~0.02 Å), exhibits an almost diagonal primary Condon locus with some indications of subsidiary loci at high v′, v″. The CN red system having a somewhat larger Δ*r_e_* (~0.05 Å) exhibits more subsidiary loci at lower *v*′, *v*″. In the tables, the power of 10 by which the entry is to be multiplied is indicated by the negative number at the end of each entry. The position of the CN “tail” bands of the violet system at the large values of *v*′, *v*″ is clearly seen on the primary locus. The data for the CN red system agrees well with that of Wyller [1958] calculated on the basis of a Hulburt-Hischfelder potential.

## 4. Discussion

While the Morse model of molecular potential is admittedly an empirical expedient, the work of Jarmain [1963] on realistic Klein-Dunham potentials for the *X*^2^Σ^+^, *A*^2^∏ and *B*^2^Σ^+^ states of CN shows that the departure from Morse potentials is small and thus that the tables 2 and 3 are probably acceptable. These Franck-Condon factors for the CN violet system have been used in conjunction with the lifetime measurements of Bennett and Dalby [1960], to determine absolute band strengths for important bands of the system [Nicholls 1964].

## Figures and Tables

**Table 1:** Basic data

State	*ω_e_* (*cm*^−1^)	*ω_e_x_e_*(cm^−1^)	*r_e_*(*A*)	*μ*_A_	*ν*_max_
					
*X*^2^Σ^+^	2068.614	13.114	1.17198	6.46427	18
*A*^2^II*_i_*,	1812.33	12.61	1.2296	6.46427	19
*B*^2^Σ^+^	2163.9	20.2	1.1493	6.46427	19

**Table 2:** Franck-Condon factors to high vibrational quantum numbers for the *CN* red (*A^2^II_i_*–*X^2^Σ^+^*) system

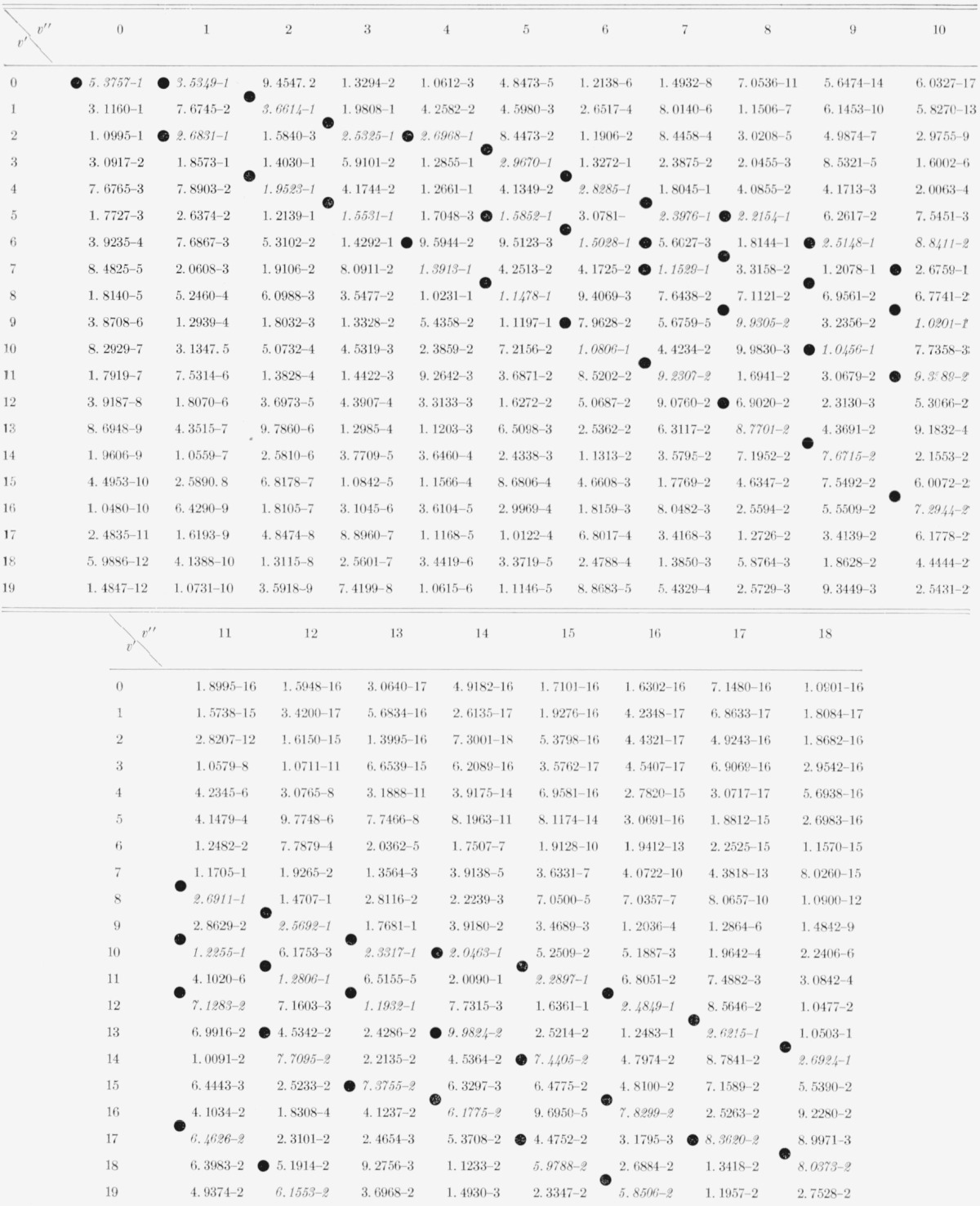

**Table 3:** Franck-Condon factors to high vibrational quantum numbers for the *CN* violet (*B^2^Σ^+^*–*X^2^Σ^+^*) system

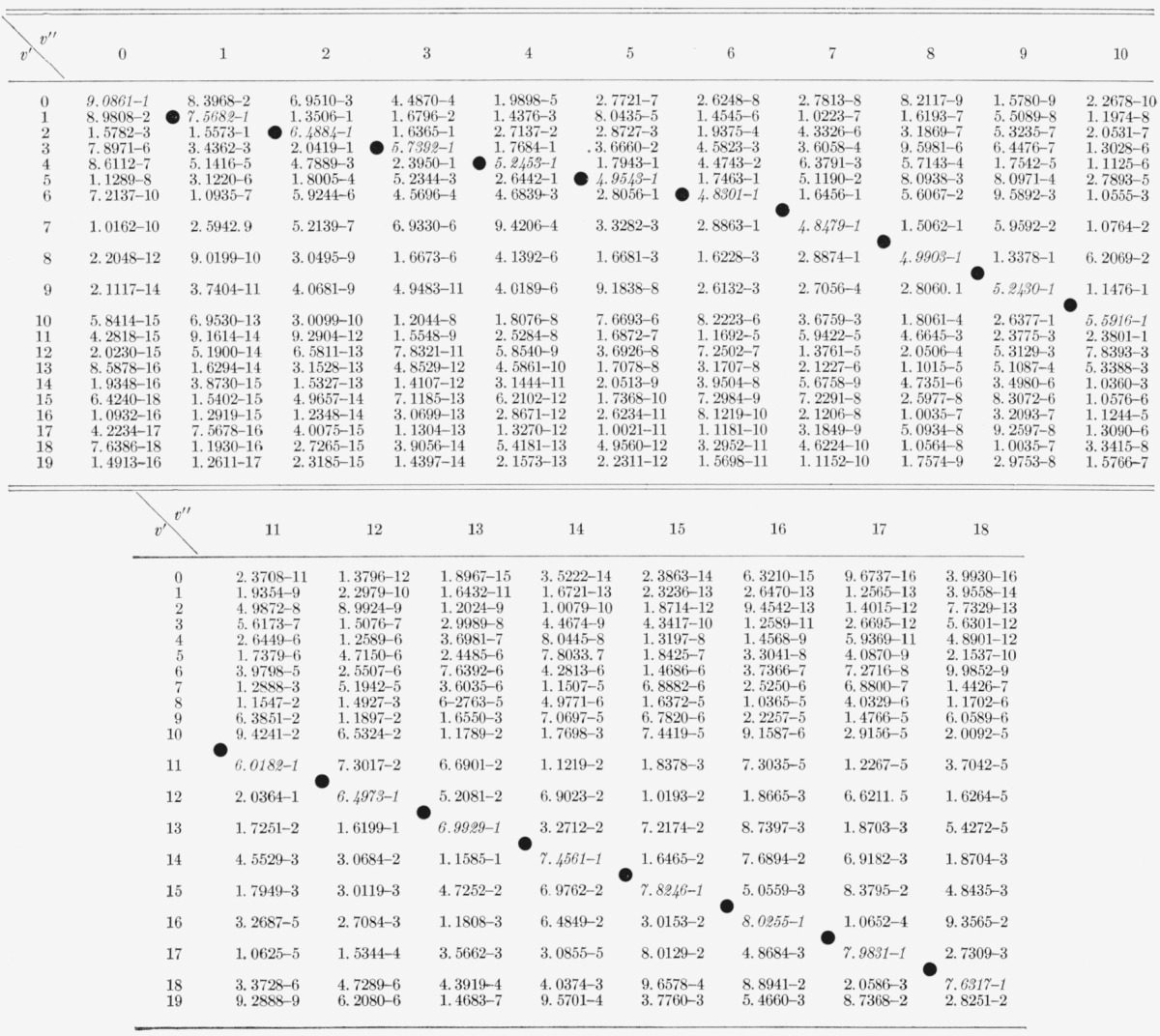
